# Cold atmospheric plasma enhances morphological and biochemical attributes of tomato seedlings

**DOI:** 10.1186/s12870-024-04961-5

**Published:** 2024-05-18

**Authors:** Sadoun M. E. Sultan, Ahmed Fathy Yousef, Waleed M. Ali, Amal A. A. Mohamed, Abdel-Raddy M. Ahmed, Mohamed. E. Shalaby, Islam I. Teiba, A. M. Hassan, Nabil A. Younes, E. F. Kotb

**Affiliations:** 1 Department of Horticulture, College of Agriculture, University of Al-Azhar (Assiut Branch), Assiut, 71524 Egypt; 2 Botany Department, Faculty of Science, Aswan University, Aswan, 81528 Egypt; 3 Department of Agronomy (Biochemistry), Faculty of Agriculture, Al-Azhar University (Assiut Branch), Assiut, 71524 Egypt; 4 Department of Plant production, Collage of Agriculture (Saba Basha), Alexandria University, Alexandria, 21531 Egypt; 5 Microbiology, Botany Department, Faculty of Agriculture, Tanta University, Tanta, 31527 Egypt; 6 Department of Physics, College of Science, University of Al-Azhar (Assiut Branch), Assiut, 71542 Egypt

**Keywords:** Carbohydrates, Non-enzymatic antioxidants, Pigment contents, Total antioxidant capacity, Total protein

## Abstract

Cold atmospheric plasma (CAP) is a physical technology with notable effects on living organisms. In the present study, tomato seeds (*Solanum lycopersicum* var. Bassimo Mill.) were exposed to CAP for various time intervals, ranging from 1 to 5 min, in both continuous and intermittent periods, and were compared with a control group that received no CAP treatment. Seedlings grown from treated seeds exhibited improvements in levels of growth traits, photosynthetic pigments, and metabolite contents when compared to the control group. Seedlings from seeds treated with S04 displayed significant increases in shoot and root lengths, by 32.45% and 20.60% respectively, compared to the control group. Moreover, seedlings from seeds treated with S01 showed a 101.90% increase in total protein, whereas those treated with S02 experienced a 119.52% increase in carbohydrate content. These findings highlight the substantial improvements in growth characteristics, photosynthetic pigments, and metabolite levels in seedlings from treated seeds relative to controls. Total antioxidant capacity was boosted by CAP exposure. The activities of enzymes including superoxide dismutase, catalase, and peroxidases were stimulated by S02 and exceeded control treatment by (177.48%, 137.41%, and 103.32%), respectively. Additionally, exposure to S04 increased the levels of non-enzymatic antioxidants like flavonoids, phenolics, saponins, and tannins over the control group (38.08%, 30.10%, 117.19%, and 94.44%), respectively. Our results indicate that CAP-seed priming is an innovative and cost-effective approach to enhance the growth, bioactive components, and yield of tomato seedlings.

**Supplementary Information:**

The online version contains supplementary material available at 10.1186/s12870-024-04961-5.

## Introduction

Tomato (*Solanum lycopersicum* Mill.) is a highly cultivated vegetable crop globally. It ranks as the third-largest vegetable crop, following potatoes and sweet potatoes. As a processing crop, it holds the top rank among all vegetables [1]. Therefore, it is crucial to implement effective cultivation management approaches for tomatoes, focusing on enhancing microbial resistance [2], environmental stress tolerance [3], yield [4], and the content/activity of active biomolecules [5]. The need to boost plant productivity has become an urgent priority in the last decades, leading researchers and producers worldwide to embrace eco-friendly innovative methods for crop cultivation. Diverse techniques have arisen to enhance plant growth, encompassing the utilization of synthetic plant protectants, the introduction of beneficial microbes into the soil, the application of manure, and the integration of nanotechnology. Additionally, the utilization of cold atmospheric plasma (CAP) is an emerging strategy aimed at improving plant growth and overall productivity.

In recent years, the use of CAP, also known as low-temperature plasma (LTP) or non-thermal plasma (NTP), has gained significant recognition in the field of biology [6]. It has a wide range of effects, including improving seed germination, enhancing growth, sterilizing surfaces, eradicating microorganisms, facilitating food production and processing, accelerating wound healing, and extending the shelf life of perishable goods [7]. In agriculture, plasma agriculture or plasma farming involves the comprehensive integration of plasma throughout the entire crop lifecycle, from pre-cultivation stages to the eventual consumption of the produce. In the field of plant sciences, scientific research on plasma treatment has predominantly focused on identifying potential, standardizing treatment protocols, and characterizing the biochemical impacts of plasma on plants [8]. Recent efforts have delved into the molecular mechanisms underlying the effects of plasma on seed germination and plant development at the cellular level. These studies involve the scrutiny of gene expression patterns, comprehensive analysis of the transcriptome, examination of protein expression profiles, and exploration of epigenetic influences [9, 10]. Seed priming has emerged as a well-established method to amplify the efficiency of seed germination and foster robust plant growth [11]. This technique involves pretreating seeds, leading to a reduction in germination time and an augmentation in vigor, resulting in higher seedling survival rates. Direct exposure of seeds to plasma induces alterations in the seedcoat structure, provoking the activation of seed germination processes, shortened germination durations, heightened resistance to diseases, and rapid progress in growth and maturation [12]. Notably, studies have demonstrated that CAP bolsters seed germination efficiency, elevates seed sterilization efficacy, and stimulates long-term growth by promoting seedling biomass, antioxidants production, plant hormone synthesis, and the expression of genes linked to defense and drought resilience [13, 14]. Recent research focusing on ginseng showcases how plasma treatment augments germination rates, sterilization effectiveness, and curbs the growth of microorganisms, particularly displaying antifungal properties that mitigate root rot [15].

DBD, a distinctive form of produces diffuse plasma layers visually at atmospheric pressure in practical gases such as N_2_, O_2_, and Ar, and has demonstrated numerous applications, particularly in the agricultural domain [16].

In this study, we aimed to investigate the potential of CAP to enhance various growth-related aspects, including growth characteristics, photosynthetic responses, total soluble proteins, antioxidant enzymes, in vitro antioxidant activity, and non-enzymatic antioxidants.

## Materials and methods

### Cold atmospheric plasma design and treatments

**Fig. 1:**
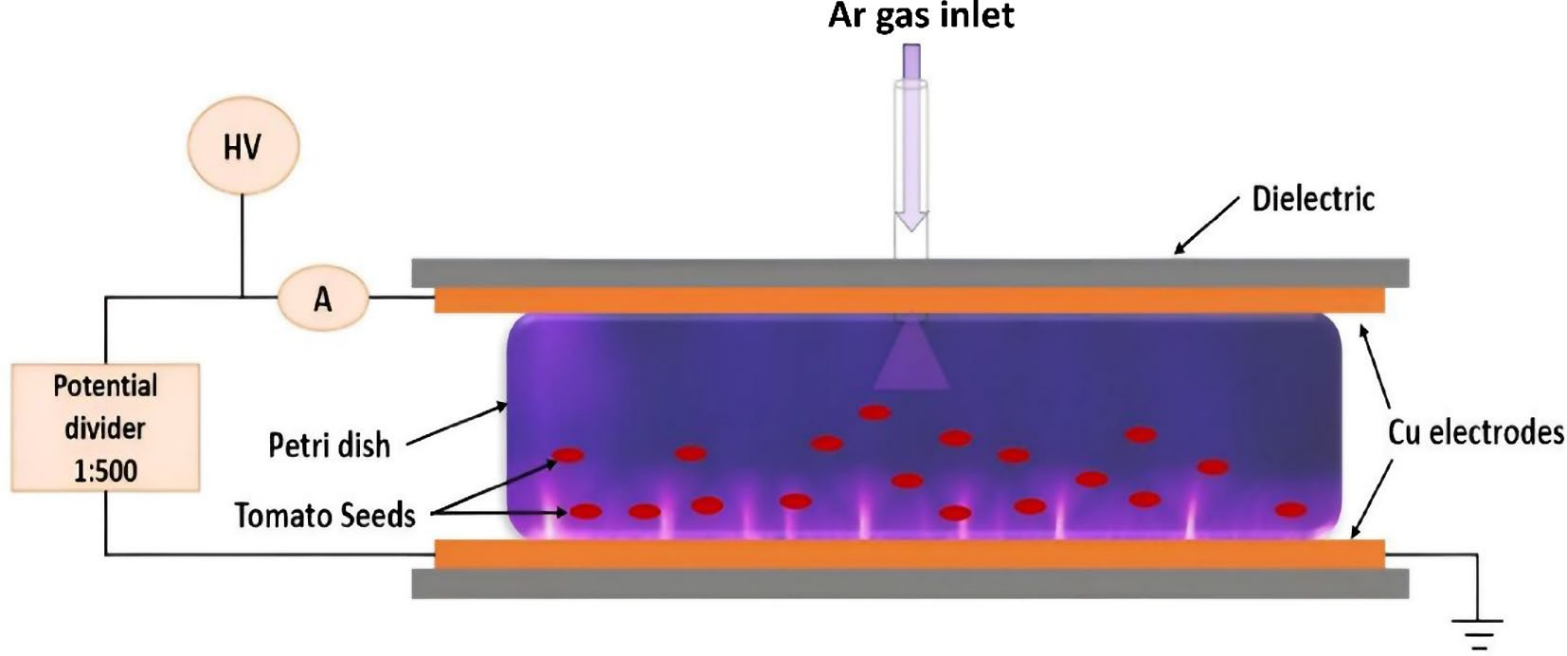
Schematic representation of the device used to generate Cold Atmospheric Plasma (CAP) through Dielectric Barrier Discharge (DBD) for treating tomato seeds. Where Ar is Argon and Cu is Copper

Tomato seeds (*Solanum lycopersicum* var. Bassimo; Monsanto Holland BV Co., Holland) were subjected to plasma treatment utilizing a Dielectric Barrier Discharge (DBD) arrangement operating with Argon (Ar) gas at ambient conditions. The DBD system used in the experiment comprised parallel disc-shaped copper (Cu) electrodes, measuring 3 mm in thickness, 50 mm in radius, and possessing a 2 mm dielectric layer. This electrode configuration was energized by AC high voltage reaching up to 20 kV peak-to-peak, facilitated by a 1:500 potential divider, while a digital multimeter gauged the discharge voltage and current. The effective surface area of the 3 mm thick DBD plasma layer spanned 10 cm × 10 cm. Within an Ar gas atmosphere, the plasma produced by DBD — hereafter referred to as ‘Ar-CAP’ — presented a macroscopically diffuse appearance. Despite being a ‘cold’ plasma, it was characterized by its remarkably high power density. At atmospheric pressure, within the presence of ambient Ar gas, plasma treatment was executed utilizing an input power of 300 W. To ensure consistent and uniform treatment, seeds were manually stirred. This methodology was specifically designed to guarantee the ongoing and consistent plasma treatment of the seeds. Varied durations for plasma treatment were established empirically for each individual species, guided by germination test outcomes (as detailed in the Results section). The seed samples were divided into two segments that underwent different types of treatment periods: continuous and intermittent. The continuous treatments involved exposing seeds to continuous CAP plasma for varying durations, identified by the following labels: S0 for the control group, S1 for 1 min, S2 for 2 min, S3 for 3 min, S4 for 4 min, and S5 for 5 min. The intermittent treatments consisted of treating seeds with CAP plasma at intermittent intervals, labeled as follows: S01 for 1 min (comprising half a minute of CAP exposure, a half-minute pause, and another half-minute of CAP exposure), S02 for 2 min (comprising one minute of CAP exposure, a minute pause, and another minute of CAP exposure), S03 for 3 min, S04 for 4 min, and S05 for 5 min, with similar interval patterns as in S01 and S02 treatments. Further elaboration on the DBD technology and plasma diagnostics is provided in Fig. 1.

### Greenhouse experiment

An experiment was conducted under controlled conditions in private greenhouse at Asfoon Village, Esna City, Luxor Governorate, Egypt (N 25.30222409140631, E 32.4866372411133) from 1 December 2022 to 25 January 2023 (56 days). The environmental conditions, including temperature and relative humidity, that occurred inside the greenhouse during the experiment are depicted in Figure S1 (refer to Supplementary data). Plasma-treated tomato seeds, as well as the control group, were planted in 209-cell plug trays (40D x 80 W x 7 H cm; Green Co., Egypt) that was filled with commercial growing substrate (3 cocopeat: 1 vermiculite; V:V), pH 5.5_6.5, Green House Egypt Co., Egypt). The commercial growing substrate was mixed with fertilizer (N20:P20:K20; Green House Egypt Co., Egypt) when preparing for cultivation. One tray (209 cells) was used for each treatment, divided into 3 replicates. By observing the moisture content in the rooting medium, suitable quantities of water were administered to the pots to ensure the plants received the necessary hydration. One week following sowing, the seedlings were initiated on a fertilization regimen using water-soluble fertilizers. The fertilizers used in the study included compound fertilizers labeled as “N20: P20: K20 + TE” from Green House Egypt Co. in Egypt. Additionally, Stimufol Amino compound fertilizers, which consisted of “N 25%, P 16%, K 12%, Amino acids 2%, Bo 0.044%, Fe 0.17%, Mo 0.001%, Zn 0.03%, Cu 0.085%, Co 0.01%, Mn 0.02%, Mg 0.085%, and EDTA,” were provided by Shoura Co. in Egypt.

### Laboratory analysis

#### Morphological variables

Growth parameters for tomato seedlings were assessed at 55 days after sowing (DAS). Plant height and root length (cm) were measured using a ruler, with measurements taken from the base of the plant at the soil surface to the highest point of the plant. Stem diameter (mm) was measured using a digital Vernier caliper. Leaf area was determined following the method described by Pandey and Singh [17].

#### Chemicals and equipment

We utilized high-performance liquid chromatography (HPLC) grade solvents and reagents for all our experiments. Spectrophotometric measurements were conducted using a Shimadzu UV-Visible Spectrophotometer with the model name UVmini-1240, manufactured by Shimadzu Corporation in Japan.

For total soluble carbohydrate content, fresh tomato leaves were individually ground into pieces using a mortar and pestle. From the milled samples, 0.5 g were extracted in triplicate using boiling ethanol with a concentration of 70% (v/v) with refluxing for 30 min. Subsequently, the extracts were filtered, and the filtrated extract was stored at 4 °C in a dark environment. The determination of total soluble carbohydrate content in tomato leaves followed the method described by DuBois et al. [18]. In summary, 1 mL of a 5% phenol solution and 5 mL of concentrated H_2_SO_4_ were mixed with 100 µL of each leaf extract. This mixture was then placed in a water bath at 30 °C for 20 min. The absorbance was measured at a wavelength (λ) of 490 nm against a blank containing distilled H_2_O. The total carbohydrate content was calculated as milligrams per gram of fresh weight (mg g^− 1^ FW) using a calibration curve for glucose as a standard, with the equation y = 1.0248x + 0.0195 (r^2^ = 0.9941).

For Photosynthetic pigments, fresh leaves (0.1 g each) were homogenized in triplicate using 2 mL of 80% acetone. The absorbance of the combined extracts was measured using a spectrophotometer at two specific wavelengths, 663 nm and 645 nm, as per the method described by Mackinney [19]. To determine the contents of chlorophyll a (Chl *a*) and chlorophyll b (Chl *b*), the following equations were employed:

1 $$\text{C}\text{h}\text{l} a (\text{m}\text{g}/\text{g} \text{F}\text{W})=\left(=\left[\right(12.7 \times \text{A}663)-(2.6 \times \text{A}645\left)\right]\text{X}\text{V}\right)$$

2 $$\text{C}\text{h}\text{l} b (\text{m}\text{g}/\text{g}\text{F}\text{W})=\left(=\left[\right(22.9 \times \text{A}645)-(4.68 \times \text{A}663\left)\right]\text{X}\text{V}\right)$$

where A663 and A645 represent the absorbance values at 663 nm and 645 nm (respectively), V is the volume of the extract (mL), and FW denotes the fresh weight of the sample (g).

For determination of total soluble proteins and antioxidant enzymes, fresh leaf samples (0.5 g each) were first ground into small pieces using a mortar that had been pre-cooled on ice. These ground samples were then homogenized in 30 mL of a 50 mM potassium phosphate buffer with a pH of 7.8. The homogenate was subsequently subjected to centrifugation at 12,000 times the force of gravity (12,000 × g) for a duration of 10 min at a temperature of 4 °C. The resulting supernatant, which is the liquid portion remaining after centrifugation, was utilized for both protein determination, as described by Lowry et al. [20], and for conducting enzyme assays as followed:

Catalase (CAT) activity was measured by recording the reduction in hydrogen peroxide (H_2_O_2_), following the method demonstrated by Díaz-Vivancos et al. [21]. The decrease in absorbance at 240 nm was monitored in a reaction mixture comprising 1.5 mL of 50 mM potassium phosphate buffer with a pH of 7.8, 40 mM H_2_O_2_, and 100 µL of the enzyme extract. CAT activity was determined using the extinction coefficient of 39.4 mM^-1^ cm^-1^. This method allows quantifying the activity of the enzyme in terms of its ability to break down hydrogen peroxide.

Peroxidase activity (POD) was assessed following the procedure outlined by Hemeda and Klein [22]. To measure POD activity, a reaction mixture was prepared containing 30 mM guaiacol, 40 mM H_2_O_2_, 50 mM potassium phosphate buffer with a pH of 6.6, and 100 µL of the enzyme extract. The enzyme activity was determined by monitoring the increase in absorbance at 470 nm, which results from guaiacol oxidation, using an extinction coefficient of 6.39 mM^-1^ cm^-1^, and the measurement was conducted over a 3-minute period. This method quantifies the activity of peroxidase by observing its ability to catalyze the oxidation of guaiacol.

The analysis of Superoxide Dismutase (SOD) activity was conducted following the method outlined by Beauchamp and Fridovich [23], with some modifications. In the assay, a mixture of 3 mL was prepared, consisting of 50 mM phosphate buffer with a pH of 7.8, 9.9 mM L-methionine, 84 mM nitroblue tetrazolium (NBT), 53 µM riboflavin, and 100 µL of the enzyme extract. The reduction of NBT through a photochemical reaction was measured at a wavelength of 560 nm. An inhibition curve was plotted against various volumes of the enzyme extract. One unit of SOD activity was defined as the volume of extract required to cause a 50% inhibition of the photochemical reduction of NBT. This method quantifies SOD activity by measuring the enzyme’s ability to inhibit the reduction of NBT in the presence of the photochemical reaction components.

For determination of the in vitro antioxidant activity, dried plant specimens weighing 100 mg were immersed in 10 mL of 80% methanol and subjected to vortexing for 1 min. Subsequently, this mixture was incubated in a water bath at 60 °C for 1 h. Afterward, the extracts underwent centrifugation at 800 rpm for a duration of 10 min, and the resulting supernatants were employed for conducting antioxidant assessments and the determination of various secondary metabolites, which are non-enzymatic antioxidants.

The assessment of free radical scavenging activity (RSA) was conducted through the utilization of the 2,2-Diphenyl-1-picryl-hydrazyl (DPPH) assay. Various concentrations of plant extracts were prepared. One milliliter of each concentration was mixed with 2 milliliters of a DPPH solution at a concentration of 0.1 mM. The samples were then left to incubate in the dark for 20 min, after which their absorbance was measured at 517 nm. The percentage of DPPH radical scavenging was determined using the formula proposed by Wang and Mazza [24]:

3 $$\text{D}\text{P}\text{P}\text{H}\, \text{R}\text{a}\text{d}\text{i}\text{c}\text{a}\text{l}\, \text{S}\text{c}\text{a}\text{v}\text{e}\text{n}\text{g}\text{i}\text{n}\text{g}\, \text{A}\text{c}\text{t}\text{i}\text{v}\text{i}\text{t}\text{y}\, \text{\%} = \left((\text{A}\text{c} - \text{A}\text{s})|(\text{A}\text{c} \times 100)\right)$$

where Ac denotes the control absorbance of the DPPH radical in methanol, and As refers to the absorbance of the DPPH radical when combined with the plant extract sample.

The IC50 value, indicating the concentration required to inhibit 50% of DPPH activity, was determined to assess the antioxidant efficacy of the plant extract.

The determination of total antioxidant capacity (TAC) was carried out using the phosphomolybdenum complex assay, a method outlined by Prieto et al. [25]. In this procedure, 1 mL of a reagent solution consisting of [0.6 M sulfuric acid, 28 mM sodium phosphate, and 4 mM ammonium molybdate] was combined with 1 mL of the plant extract. The resulting mixture was incubated at 90 °C for a duration of 90 min. Subsequently, the absorbance of the mixture was measured at 700 nm against a blank. Ascorbic acid served as the standard reference, and the total antioxidant capacity (TAC) was expressed as micrograms of ascorbic acid (AA) equivalent per milligram of DW.

The quantification of the total flavonoid content was conducted using the aluminum chloride colorimetric method, as described by [26]. In summary, 1 mL of the plant extract was combined with 300 µL of NaNO_2_ (5% w/v). After 6 min, 300 µL of AlCl_3_ (10% w/v) was introduced to the mixture. Following a 6-minute incubation period, 400 µL of NaOH (1 M) was added. The absorbance was then measured at 510 nm. Quercetin was used as the reference standard, and the total flavonoid content was expressed as milligrams of quercetin equivalent per gram of DW.

The quantification of the total saponins, 1 mL of each extract was combined with 5 mL of a vanillin reagent prepared in H_2_SO_4_ at a concentration of 2%. Purified saponin served as the sample, and both the samples and the standard were subjected to incubation at 60 °C for one hour. Subsequently, they were placed in ice for 10 min to cool. The absorbance of the samples was then measured at 473 nm, following the method outlined by Ebrahimzadeh [27]. The saponin content was determined and expressed as milligrams of saponins equivalent per gram of DW.

We determined the total tannin content by employing the vanillin assay method, as outlined by Sun et al. [28]. The procedure involved mixing 1 mL of each extract with Vanillin reagent, which was prepared at a concentration of 4% in methanol. Subsequently, 1 mL of concentrated HCl was introduced into the mixture, which was then left to incubate at room temperature for 20 min. A similar treatment was applied to the catechol standard. The absorbance of both the samples and the standard was measured at a wavelength of 550 nm. The total tannin content was expressed in terms of milligrams of catechol equivalent per gram of DW.

The total phenolic content in the samples was determined using a modified Folin-Ciocalteu method, following the protocol established by Singleton et al. [29]. This involved mixing 1 mL of each extract with 4 mL of 2% Na_2_CO_3_, followed by the addition of 5 mL of diluted Folin-Ciocalteu reagent. After an incubation period of 1 h at room temperature, the absorbance was measured at 700 nm. The phenolic content was calculated using gallic acid as the reference standard and expressed in milligrams of gallic acid equivalent per gram of DW. This method provided a means to quantify the total phenolic content in the samples accurately.

### Statistical analysis

Significant differences among various cadmium treatments were identified by conducting a one-way analysis of variance (ANOVA) using Statistix version 8.1. A difference was considered significant when the *p-value* was less than 0.05. To categorize the distinct patterns of growth attributes, enzymatic and non-enzymatic antioxidants in response to CAP, a Principal Component Analysis (PCA) was conducted using XLSTAT version 2016.3. The correlation matrix was then visualized using Excel 365. The experiments were carried out with three biological replicates (*n* = 3).

## Results

### Effects of plasma on seedling growth morphological variables

The results of the study indicate that the application of CAP had a significant impact on the morphology of tomato seedlings (Table 1; see Supplementary data for Table S1 and Figure S2). Seedlings from seeds treated with S04 reached the greatest height at 22.92 cm, without showing significant differences when compared to S02 and S03, which recorded heights of 22.23 cm and 22.41 cm, respectively. Similarly, seedlings from S04-treated seeds exhibited the longest root length at 9.50 cm, but this did not significantly differ from seedlings originating from seeds treated with S05 and S01, with root lengths of 8.13 cm and 8.52 cm, respectively. Conversely, seedlings from S02-treated seeds had the shortest root length at 7.16 cm. As for stem diameter, seedlings from S02-treated seeds displayed the largest diameter at 0.45 cm, while those from S03-treated seeds had the smallest diameter at 0.31 cm. Additionally, seedlings from seeds treated with S1 and S4 produced the highest number of leaves (7), with no significant difference observed among other treatments except for the control, S03, and S04, all of which had 6 leaves. Seeds treated with S05 exhibited the largest leaf area (40.39 cm²), surpassing other treatments, whereas the control (S0) treatment resulted in the smallest leaf area (23.81 cm²).

**Table 1:** Influence of cold atmospheric plasma (CAP) on morphological variables of tomato seedlings

Treatment	Shoot length (cm)	Root length (cm)	Stem diameter (cm)	Number of leaves per plant	Leaf area (cm^2^)
**S0**	17.30 ± 0.98^b^	7.88 ± 1.13^b^	0.39 ± 0.06^ab^	6.00 ± 0.00^b^	23.81 ± 0.81^f^
**S1**	20.54 ± 4.55^ab^	7.51 ± 0.73^b^	0.34 ± 0.08^b^	7.00 ± 0.00^a^	35.24 ± 2.00^b^
**S2**	22.23 ± 3.20^a^	8.08 ± 1.33^b^	0.37 ± 0.12^ab^	6.67 ± 0.58^ab^	26.66 ± 1.04^e^
**S3**	20.08 ± 0.28^ab^	7.19 ± 0.49^b^	0.31 ± 0.02^b^	6.33 ± 0.58^ab^	30.16 ± 1.16^d^
**S4**	22.41 ± 0.57^a^	7.44 ± 0.31^b^	0.33 ± 0.05^b^	7.00 ± 1.00^a^	32.86 ± 2.00^c^
**S5**	20.67 ± 1.27^ab^	8.13 ± 0.67^ab^	0.41 ± 0.06^ab^	6.67 ± 0.58^ab^	26.40 ± 0.40^e^
**S01**	19.21 ± 2.03^ab^	8.52 ± 0.97^ab^	0.39 ± 0.05^ab^	6.33 ± 0.58^ab^	33.58 ± 0.58^c^
**S02**	19.79 ± 1.37^ab^	7.16 ± 0.73^b^	0.45 ± 0.05^a^	6.67 ± 0.58^ab^	24.21 ± 1.21^f^
**S03**	17.71 ± 3.03^b^	8.08 ± 0.76^b^	0.36 ± 0.09^ab^	6.00 ± 0.00^b^	30.00 ± 2.00^d^
**S04**	22.92 ± 1.78^a^	9.50 ± 0.40^a^	0.40 ± 0.04^ab^	6.00 ± 0.00^b^	30.15 ± 0.15^d^
**S05**	19.33 ± 1.78^ab^	7.97 ± 0.98^b^	0.41 ± 0.02^ab^	6.67 ± 0.58^ab^	40.39 ± 2.00^a^

The values presented in the results are means ± standard deviations (SDs) of three biological replicates (*n* = 3). The data were subjected to Duncan’s multiple range test at a significance level of *p* = 0.05. Groups of means within the same column that share the same letters were considered not to have significant differences. For details of the treatments, you can refer to the material section

### Effects of plasma on carbohydrates, photosynthetic pigments, and soluble protein contents in seedlings

Plants of tomato differently responded to various levels of CAP in comparison to control (Table 2 and Table S1). Plants subjected to S03 had the highest carbohydrate content (23.65 mg g^− 1^ FW) among all treatments where they accumulated about 2-fold than the control plants. In addition, control plants exhibited the lowest content of carbohydrates in comparison to other treatments (Table 2). Exposure of plants to CAP from S2 to S4 enhanced the production of total chlorophylls with the highest content of total chlorophylls in plants subjected to S3 (Table 2). Also, Protein production was enhanced in response to high levels of CAP. The content of protein was duplicated in plants subjected to S01 in comparison to control plants (Table 2).

**Table 2:** Influence of cold atmospheric plasma (CAP) on the content of different primary metabolites in the leaves of tomato seedlings

Treatment	Carbohydrates (mg g^− 1^ FW)	Chlorophyll (µg mg^− 1^ FW)		Total chlorophyll	Total protein (mg g^− 1^ FW)
		Chl a	Chl b		
**S0**	7.35 ± 0.17^i^*	2.65 ± 0.02^g^	1.91 ± 0.33^fg^	4.56 ± 0.30^h^	34.32 ± 0.39^g^
**S1**	9.46 ± 0.49^h^	3.92 ± 0.01^d^	2.65 ± 0.06^bcd^	6.57 ± 0.07^d^	37.96 ± 4.95^fg^
**S2**	19.26 ± 0.62^c^	5.03 ± 0.01^b^	2.91 ± 0.03^abc^	7.94 ± 0.02^b^	42.81 ± 4.37^def^
**S3**	17.71 ± 0.00^d^	5.90 ± 0.01^a^	3.07 ± 0.01^ab^	8.97 ± 0.01^a^	35.11 ± 2.70^g^
**S4**	17.74 ± 0.12^d^	4.86 ± 0.24^bc^	2.40 ± 0.11^de^	7.26 ± 0.13^c^	38.79 ± 1.03^efg^
**S5**	11.86 ± 0.17^f^	3.41 ± 0.01^e^	2.16 ± 0.07^ef^	5.57 ± 0.06^f^	21.86 ± 0.05^h^
**S01**	10.74 ± 0.08^g^	2.76 ± 0.03^fg^	2.83 ± 0.16^cde^	5.59 ± 0.24^f^	69.28 ± 0.39^a^
**S02**	16.13 ± 0.08^e^	4.58 ± 0.06^c^	3.30 ± 0.41^a^	7.88 ± 0.35^b^	56.59 ± 0.88^b^
**S03**	23.65 ± 0.12^a^	2.67 ± 0.21f^g^	1.66 ± 0.07^g^	4.43 ± 0.31^h^	42.92 ± 0.59^de^
**S04**	19.35 ± 0.00^c^	2.96 ± 0.01^ef^	2.19 ± 0.01^fg^	5.15 ± 0.01^g^	47.22 ± 1.37^cd^
**S05**	22.04 ± 0.00^b^	3.90 ± 0.01^d^	2.07 ± 0.00^ef^	5.97 ± 0.01^e^	50.10 ± 0.15^c^

The values presented in the results are means ± standard deviations (SDs) of three biological replicates (*n* = 3). The data were subjected to Duncan’s multiple range test at a significance level of *p* = 0.05. Groups of means within the same column that share the same letters were considered not to have significant differences. For details of the treatments, you can refer to the material section

### Effects of plasma on antioxidant enzyme activity in seedlings

**Fig. 2:**
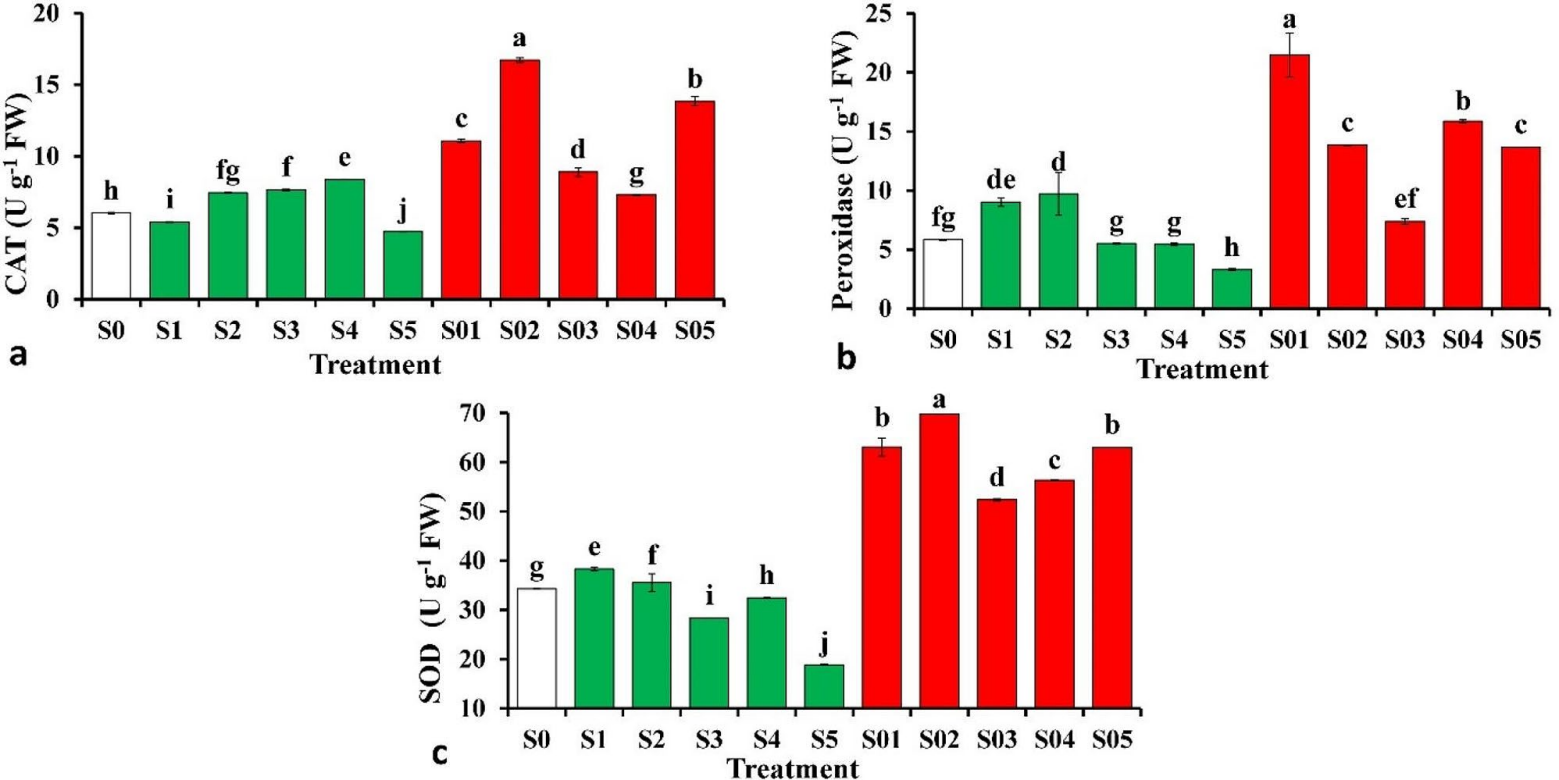
Influence of cold atmospheric plasma (CAP) on activities of antioxidant enzymes catalase (a), peroxidase (b) and superoxide dismutase (c) of tomato seedlings. Values are means ± standard deviations (SDs) of data obtained from three biological replicates (*n* = 3). Following the application of Duncan’s multiple range test at a significance level of *p* = 0.05, it was observed that means within the same column that share identical letters were not significantly different from each other. For details of the treatments, you can refer to the material section

Significant differences among different levels of CAP treatments were recorded in the antioxidant enzyme activities (Fig. 4 and Table S1). Activity of SOD (Fig. 2a) was much higher plants subjected to S02 than that of control. Activity of POD (Fig. 2b) and CAT (Fig. 2c) increased in plants subjected to CAP with highest values in S01 and S02, respectively.

Two assays were used for measuring antioxidants. First, by measuring IC50 using DPPH assay, the lowest value of IC50 is the highest antioxidant activity (radical scavenging). Plants subjected to CAP (S4 to S04) exhibited the highest radical scavenging activity with low values of IC50 (Table 3). Second, TAC was measured by phosphomolybdate assay; it was significantly increased by increasing (S0) CAP where plants subjected to S01 exhibited the highest TAC (Table 3).

**Table 3:** Influence of cold atmospheric plasma (CAP) on the values of IC50 (mg mL^− 1^) and total antioxidant capacity [(TAC), µg ascorbic acid equivalent mg^− 1^ DW] of the leaves of tomato seedlings

Treatment	Antioxidant activity	
	IC50	TAC
**S0**	4.59 ± 0.01^a^	1699 ± 225^fg^
**S1**	2.05 ± 0.28^c^	1256.6 ± 50.1^gh^
**S2**	2.56 ± 0.05^b^	980.1 ± 59.4^h^
**S3**	2.01 ± 0.01^c^	1531 ± 150^g^
**S4**	0.81 ± 0.01^e^	2442 ± 726^de^
**S5**	0.87 ± 0.14^e^	2727.9 ± 46.9^cd^
**S01**	0.75 ± 0.01^e^	4157.1 ± 65.7^a^
**S02**	0.83 ± 0.08^e^	3628.3 ± 25.0^ab^
**S03**	0.84 ± 0.02^e^	2305.3 ± 106.4^de^
**S04**	0.89 ± 0.02^e^	3248 ± 150^bc^
**S05**	1.73 ± 0.01^d^	2188.1 ± 34.4^ef^

The values presented in the results are means ± standard deviations (SDs) of three biological replicates (*n* = 3). The data were subjected to Duncan’s multiple range test at a significance level of *p* = 0.05. Groups of means within the same column that share the same letters were considered not to have significant differences. For details of the treatments, you can refer to the material section. Where: IC50 = the concentration of a substance required to scavenge 50% of free radicals present in a system

### Effects of plasma on contents of non-enzymatic antioxidants in seedlings

**Fig. 3:**
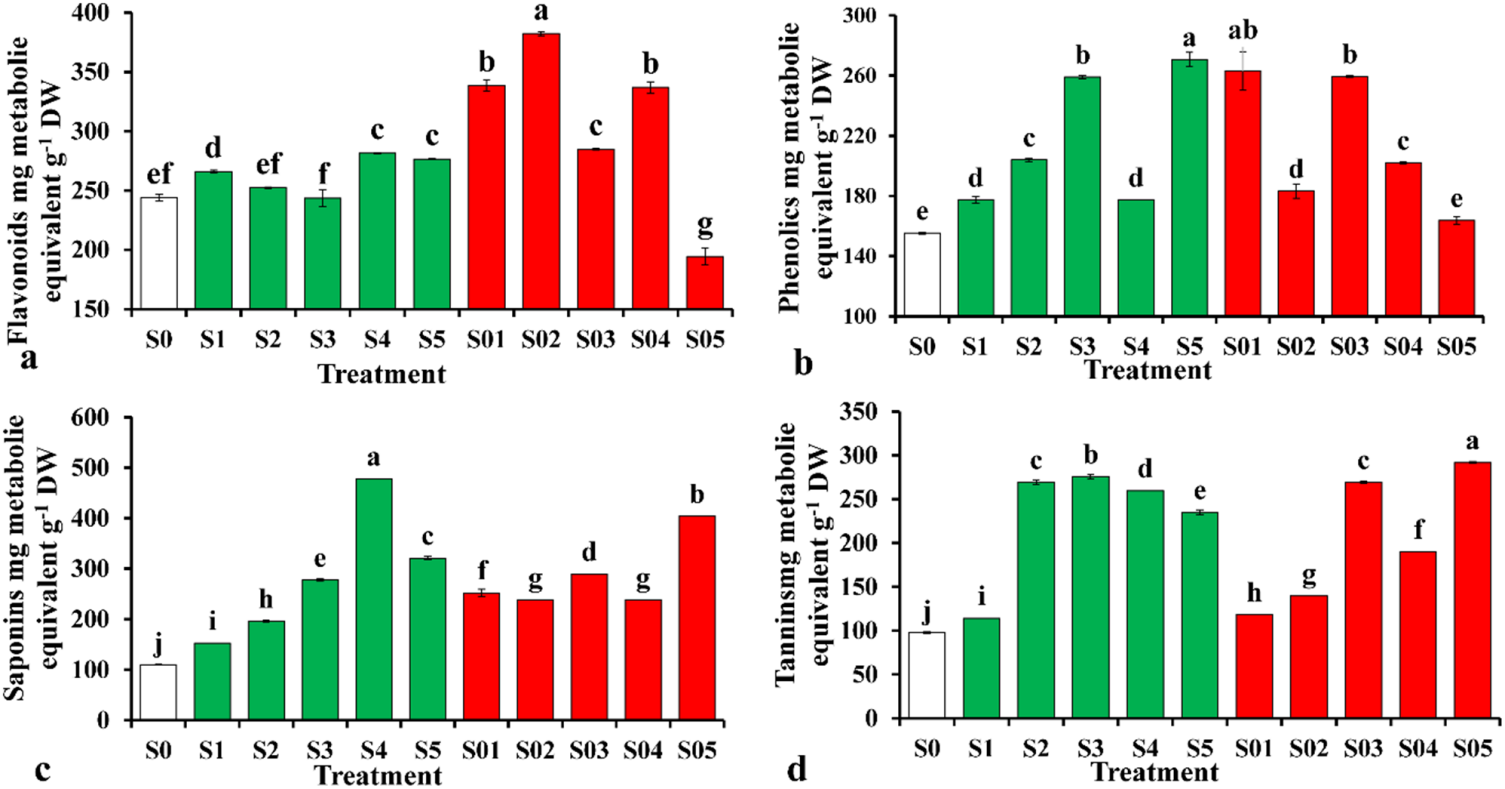
Influence of cold atmospheric plasma (CAP) on Content of different secondary metabolites (non-enzymatic antioxidants: Flavonoids (a); Phenolics (b); Saponins (c); Tannins (d)) in the leaves of tomato seedlings. Values are means ± standard deviations (SDs) of data obtained from three biological replicates (*n* = 3). Following the application of Duncan’s multiple range test at a significance level of *p* = 0.05, it was observed that means within the same column that share identical letters were not significantly different from each other. For details of the treatments, you can refer to the material section

Effect of CAP on non-enzymatic antioxidants (flavonoids, phenolics, saponins and Tannins) in the leaves of tomato was recorded (Fig. 3). They exhibited different responses regarding the levels of CAP (Table S1). In plants subjected to S02, the production of flavonoids in the leaves was significantly enhanced by 56.6% as compared to the control (Fig. 3a). At the same time, it was declined when plants subjected to the highest CAP (S05) also. Phenolics had different pattern in response to CAP; it exhibited lower contents at control plants than those subjected to CAP (Fig. 3b). Plants subjected to S4 CAP level accumulated highest content of saponins in comparing to the rest treatments, while the lowest content was recorded in control plants (Fig. 3c). Tannins accumulated in plants subjected to S05 about 2-fold more than control plants (Fig. 3d).

Details on Pearson’s correlation analyses, covering antioxidant activity, quantities of primary metabolites, antioxidant enzymes, and the content of non-enzymatic antioxidants in tomato plants exposed to varying levels of CAPs, can be found in Figure S3 (refer to Supplementary data). High positive correlation (*r* = 0.752; *p* < 0.001)) was recorded between TAC and flavonoids. Strong positive correlation was also between TAC and proteins (*r* = 0.494; *p* = 0.02). Also, antioxidant enzymes show high significant correlations (*p < 0.001*) with protein. Peroxidase, superoxide dismutase and catalase were positively correlated with protein (values of r equal to 0.911, 0.874 and 0.722, respectively). Saponins and tannins were positively correlated to carbohydrates (*r* = 0.543, 0.806; respectively).

**Fig. 4:**
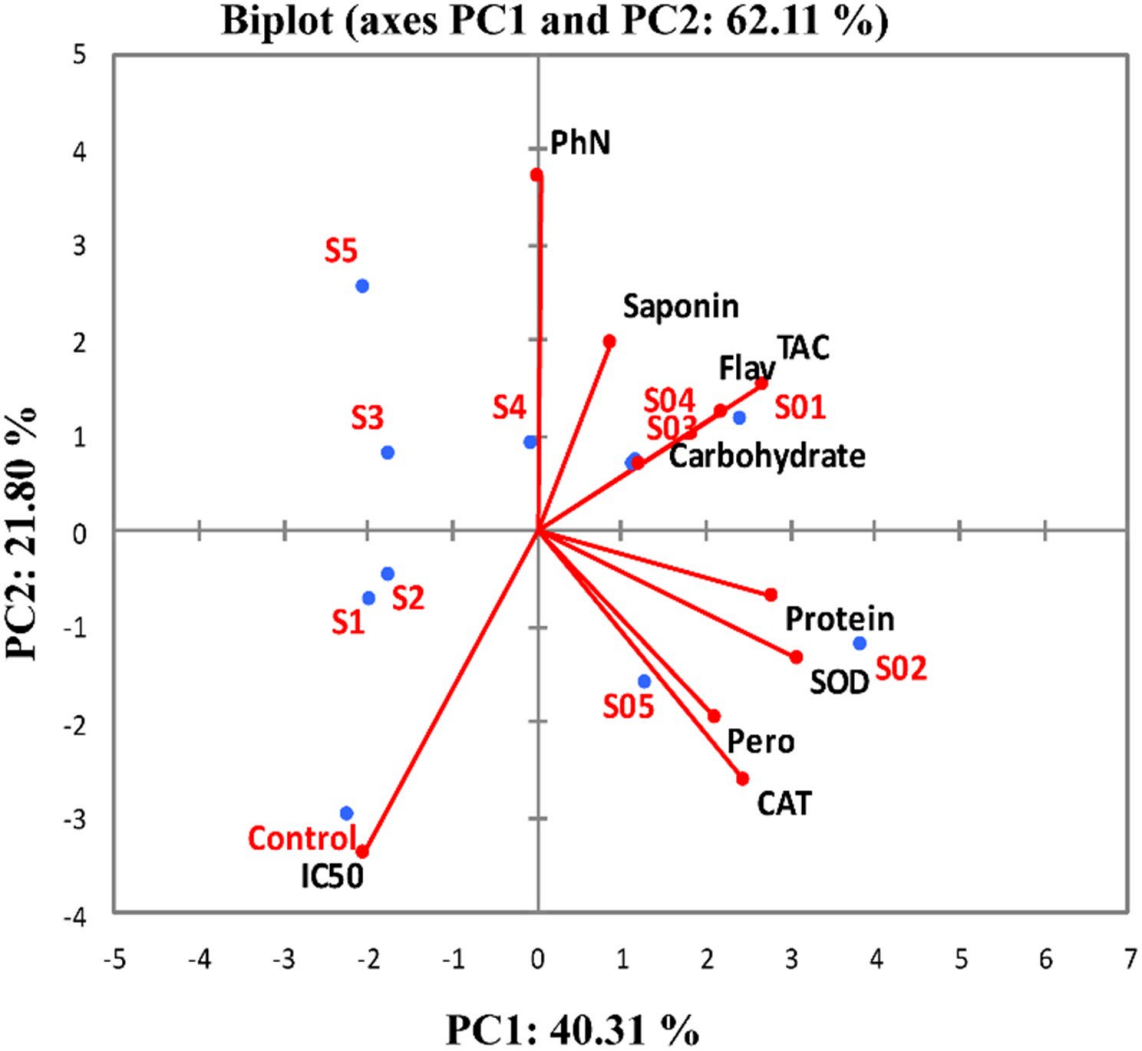
PCA Loading biplot: The non-enzymatic antioxidants in relation to antioxidant enzymes of Tomato plants subjected to different levels of CAP. CAT = catalase; FLV = total flavonoids; PHN = total phenolics; Pero = peroxidase; SAP = total saponins; SOD = superoxide dismutase; TAC = total antioxidant capacity; TAN = tannins

Figure 4 summarizes the multi correlation patterns of enzymatic and non-enzymatic antioxidants of tomato subjected to different levels of CAP using PCA analysis. The first two principal components (PCs) of PCA accounted for 62.11% of the variation in enzymatic antioxidant and non-enzymatic antioxidant traits and levels of CAP (Fig. 4). The PC1 accounted for 40.31% of the variance and was significantly positively correlated with protein content and enzyme activities (catalase, peroxidase, and superoxide dismutase). The PC2 accounted for 21.80% of the variance and was correlated positively with non-enzymatic antioxidants (total flavonoids, total, phenolics, total saponins and tannins), total antioxidant activity (TAC) and carbohydrates). Application of CAP on tomato seedlings at intermittent intervals was positively linked to the enhancement of both enzymatic and non-enzymatic antioxidants.

## Discussion

Plant encouragement is a method for achieving improved plant development and constitutes a collaborative approach utilized in contemporary and eco-conscious farming [30]. Biophysical methods like the utilization of cold atmospheric plasma exhibit remarkable potential as environmentally friendly techniques within agriculture, contributing to heightened yield and improved crop quality through a cost-effective approach [31]. The findings from this study demonstrated the beneficial effects of CAP on the growth of tomato seedlings. Moreover, the data suggested that the biological effects of treating with CAP might correlate with varying periods of exposure. Multiple investigations have indicated that seeds treated with plasma exhibit accelerated germination compared to untreated seeds. This enhanced germination outcome subsequently leads to the enhancement of seedling development, promotion of overall plant growth, and amplified yields across various crops [32–35].

Seeds exposed to S04 had the tallest shoots and roots, seeds exposed to S02 showed the thickest stems, and seeds exposed to S05 demonstrated the largest leaf area, with no significant differences among other treatments for most traits. Various reports are available that show the effect of treatment with atmospheric pressure plasma on plant seeds or in the vegetative stage for different periods of time using several working gases [16]. Ar-CAP treatment for 1 min increased the shoot and root lengths and total leaf area of *Capsicum annuum*, while 2 min of Ar-CAP treatment had the opposite effect [36]. The atmospheric pressure plasma treatment, utilizing a gaseous Air plasma jet to generate post-treatment water (PTW) plasma on tomato seeds, resulted in enhanced seedling growth [37]. Sunflower plants treated with atmospheric pressure plasma using Air scalar CAP showed improvement in seedling growth [38, 39]. The length and weight of tomato seedlings subjected to plasma treatment significantly increased compared to untreated seeds, which aligns with our findings [40]. Increased auxin levels and stimulated cell elongation due to CAP may account for the tallest plant height and longest roots observed [41, 42]. Additionally, Enhanced cell division and expansion triggered by CAP could contribute to larger leaf surfaces [41]. Moreover, altered cell wall synthesis and nutrient uptake influenced by CAP might affect stem thickness [41]. Evidence from Mildažienė et al. [43] and Ji et al. [44] points towards cold plasma altering auxin/cytokinin ratios and gibberellin levels in sunflower and wheat seeds, respectively, potentially contributing to observed growth increases. Stolárik et al. [33] provide further support with data suggesting changes in auxin and cytokinin profiles in peas, including increased IAA and zeatin, in line with enhanced growth parameters. However, the interplay between different hormones is intricate, with potential mutual regulation and “crosstalk” mechanisms adding complexity [45]. Therefore, a more comprehensive analysis of plant hormonal responses to cold plasma is crucial to unravel the precise mechanisms driving these observed plant growth transformations.

Chlorophylls, the main pigments in photosynthesis, are important for plant growth and productivity [46]. When plants are stressed, they may produce less chlorophyll, which can lead to a decrease in biomass production [47]. Our study showed that subjecting seeds to S3 significantly impacts the amount of chlorophyll *a*, while the highest level of chlorophyll *b* was attained with the application S02. Furthermore, it was observed that CAP has the capacity to enhance the levels of both chlorophyll *a* and chlorophyll *b* in plant leaves [48, 49]. CAP can also stimulate the production of ROS, which can act is signaling molecules to promote chlorophyll accumulation [50]. In this study, the content of protein was duplicated in plants subjected to S01 in comparison to control plants. Our results agreed with Zhang and his colleagues who noticed the enhanced concentration of soluble protein, in soybean after Ar-plasma treatment [51]. CAP-generated reactive species can break protein bonds and modify sulfur-containing amino acids, leading to structural changes, reduced enzyme activity, and increased solubility [52].

Zhang et al. [51], Attri et al. [16], and Abarghuei et al. [48] discovered that CAP treatment boosted the critical antioxidant enzymes and energy molecules in soybeans. These enzymes help protect plants from damage and promote growth [53]. In the present study, CAP treatment increased the antioxidant activity of plants by increasing their radical scavenging activity and total antioxidant capacity. Plants that were exposed to CAP were better able to scavenge free radicals and protect themselves from oxidative damage. Free radicals are unstable molecules that can damage cells and tissues [54]. Antioxidant compounds help to neutralize free radicals and protect cells from damage [54]. The increased antioxidant activity of irradiated plants may have several benefits, such as improved resistance to stress [55, 56], increased crop yields [57], and improved nutritional value [58].

In our study, flavonoid production was significantly enhanced by 56.6% in plants subjected to S02 but declined in plants subjected to S05. Also, phenolic content, saponins content, and tannins were highest in plants subjected to (S5, S4, and S05) CAP level, respectively, and lowest in control plants. Grzegorzewski et al. [59] studied the effects of cold plasma treatment on lettuce. They found that plasma treatment increased the production of flavonoids, beneficial compounds found in lettuce leaves. The same research found that when argon ions combine with reactive oxygen species (ROS), such as hydroxyl radicals and oxygen radicals, they can damage the epidermal cells of lettuce leaves. This damage causes the cells to release flavonoids and other compounds from the central vacuole. As a result, plasma treatment increases the levels of specific flavonoids in lettuce leaves, such as protocatechuic acid, luteolin, and diosmetin. The impact of cold plasma treatment on basil was assessed, and it was observed that the highest levels of total flavonoids were achieved with treatments of 15 kV for 20 min and 20 kV for 30 min [48]. However, the application of plasma treatment did not lead to a significant alteration in the total phenol content within basil plants [48].

Overall, this study suggests that CAP as an eco-friendly and sustainable method for enhancing crop production and quality. Nevertheless, it is essential to conduct additional research to fine-tune the CAP treatment conditions tailored to specific crops and to delve into the long-term impacts of CAP treatment on plant growth and overall development.

## Conclusion

Cold Atmospheric Plasma (CAP) significantly promoted growth and biomass in tomato seedlings. This enhancement was attributed to improved quantity of osmoregulation components, increased production of secondary metabolites, and the activation of the ROS scavenging system, which includes both enzymatic and non-enzymatic antioxidants. These processes collectively reduced lipid peroxidation, thereby improving the quality of the tomato seedlings. Furthermore, the study confirmed that CAP treatment did not lead to any deformities or adverse changes in the plants, underscoring its potential as a commercially viable technique for agriculture. These findings support the adoption of CAP treatment as an effective and environmentally friendly method for crop enhancement.

### Electronic supplementary material

Below is the link to the electronic supplementary material.

Supplementary Material 1

## Data Availability

All data available within the article.
