# Supplementary material for: Cold atmospheric plasma enhances morphological and biochemical attributes of tomato seedlings
Source: BMC Plant Biol. 2024 May 18;24:420. doi: 10.1186/s12870-024-04961-5 (PMC11102223; doi:10.1186/s12870-024-04961-5)
Supplement: Supplementary file 1 — Supplementary Material 1 [file 12870_2024_4961_MOESM1_ESM.pdf]

Supplementary data

Cold atmospheric plasma enhances morphological and biochemical attributes of tomato seedlings.

Sadoun M. E. Sultan[1,#], Ahmed Fathy Yousef [1, #], Waleed M. Ali [1,#], Amal A.A. Mohamed [2,#], Abdel- Raddy M. Ahmed [3], Mohamed. E. Shalaby [4], Islam I. Teiba [5], A. M. Hassan[6], Nabil A. Younes [1], and E. F. Kotb[6,*]

[1] Department of Horticulture, College of Agriculture, University of Al-Azhar (Assiut Branch), Assiut 71524, Egypt.

[2] Botany Department, Faculty of Science, Aswan University, Aswan 81528, Egypt.

[3] Department of Agronomy (biochemistry), Faculty of Agriculture, Al-Azar University (Assiut Branch), Assiut 71524, Egypt.

[4] Department of Plant production, Collage of Agriculture (Saba Basha), Alexandria University, Alexandria 21531, Egypt.

[5] Microbiology, Botany Department, Faculty of Agriculture, Tanta University, 31527, Tanta, Egypt

[6] Department of Physics, College of Science, University of Al-Azhar (Assiut Branch), Assiut, 71542, Egypt.

[#] Equal contribution

*Corresponding author: eizaldeen@yahoo.com

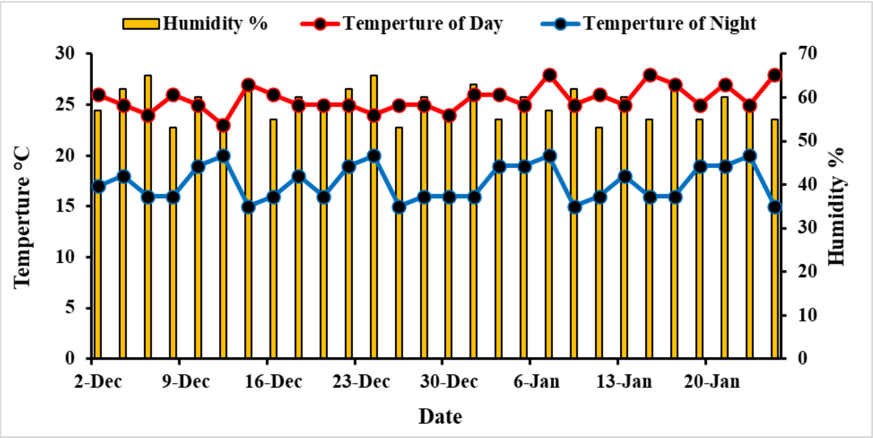

Figure S1: Environmental conditions (temperature and relative humidity) in the greenhouse during seedling growth (from December 1, 2022, to January 25, 2023).

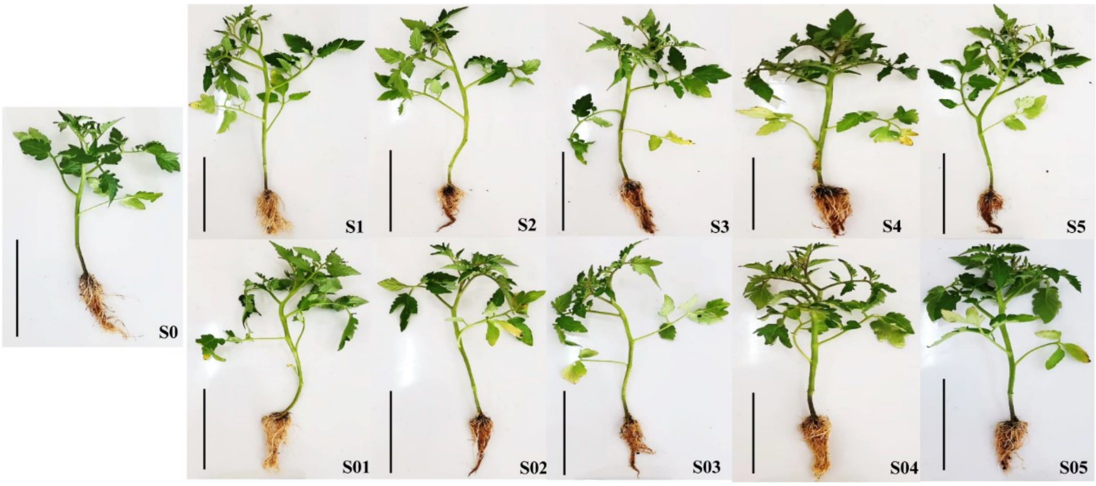

Figure S2: Influence of cold atmospheric plasma (CAP) on morphology of tomato seedlings. The scale = 10 cm according to each picture individually.

	FLV	TAC	PHN	IC50	Protein	Carbohyd	SOD	CAT	Pero	SAP
TAC	0.752									
PHN	0.199	0.263								
IC50	-0.543	-0.634	-0.472							
Protein	0.494	0.602	-0.04	-0.327						
Carbohydrate	-0.111	-0.038	0.099	-0.418	0.144					
SOD	0.467	0.593	-0.189	-0.341	0.874	0.306				
CAT	0.342	0.504	-0.201	-0.34	0.722	0.364	0.825			
Pero	0.484	0.623	-0.019	-0.32	0.911	0.051	0.833	0.566		
SAP	-0.149	0.216	0.091	-0.588	-0.013	0.543	0.011	0.276	-0.106	
TAN	-0.476	-0.301	0.254	-0.269	-0.281	0.806	-0.224	0.017	-0.343	0.68

Color legend: Significant ($P<0.05$)..non significant($p>0.05$)

Figure S3: Correlation coefficients (r) between antioxidant activity, antioxidant enzymes and non-enzymatic antioxidants. CAT= catalase; FLV= total flavonoids; PHN= total phenolics; Pero= peroxidase; SAP= total saponins; SOD= superoxide dismutase; TAC= total antioxidant capacity, TAN= tannins.

Table S1. Analysis of variance of the effect of cold atmospheric plasma (CAP) on morphological variables, chlorophyll contents, antioxidant enzyme activity, and non-enzymatic antioxidants of tomato seedlings.

	Parameters	DF	SS	MS	F-ratio	*P-value*
**Morphological variables**	**Shoot length**	10	99.653	9.96529**	1.81	0.1234
	**Root length**	10	13.2947	1.32947*	2.00	0.0895
	**Stem diameter**	10	0.05082	0.00508*	1.44	0.2349
	**Leaves number**	10	4.2424	0.42424*	1.43	0.2381
	**Leaf area**	10	754.037	75.4037**	152.17	0.0000
**Primary metabolites**	**Chlorophyll *a***	10	9.0309	0.90309*	10.19	0.0000
	**Chlorophyll *b***	10	66.7234	6.67234**	205.06	0.0000
	**Total chlorophyll**	10	570.790	57.0790**	853	0.0000
	**Carbohydrates**	10	32.0129	3.20129**	50.94	0.0000
	**Total protein**	10	3139.51	313.951**	62.5	0.0000
**Antioxidant enzyme activity**	**CAT**	10	2.752E+07	2751927 **	287	0.0000
	**Peroxidase**	10	2.009E+07	2008566**	34.1	0.0000
	**SOD**	10	268.825	26.8825**	1192	0.0000
	**IC50**	10	610.572	61.0572**	96.2	0.0000
	**TAC**	10	5628.35	562.835**	16636	0.0000
**Non-enzymatic antioxidants**	**Flavonoids**	10	56091.5	5609.15**	388	0.0000
	**Phenolics**	10	38825.6	3882.56**	193	0.0000
	**Saponins**	10	223388	22338.8**	3220	0.0000
	**Tannins**	10	113139	11313.9**	5307	0.0000

Where: DF= degree of freedom; SS = Sum of squares; MS = Mean squares; IC50 = the concentration of a substance required to scavenge 50% of free radicals present in a system; TAC = total antioxidant capacity.
